# 3D printable SiO_2_ nanoparticle ink for patient specific bone regeneration†

**DOI:** 10.1039/c9ra03641e

**Published:** 2019-07-31

**Authors:** Uday Kiran Roopavath, Raghav Soni, Urbashi Mahanta, Atul Suresh Deshpande, Subha Narayan Rath

**Affiliations:** Regenerative Medicine and Stem Cell (RMS) Lab, Department of Biomedical Engineering, Indian Institute of Technology Hyderabad (IITH) Kandi (V), Sangareddy (M), Medak-502285 Telangana India subharath@iith.ac.in +91-40-2301-6032 +91-40-2301-7111; Department of Material Science and Metallurgical Engineering, Indian Institute of Technology Hyderabad Kandi, Medak-502285 Telangana India

## Abstract

Sodium alginate and gelatin are biocompatible & biodegradable natural polymer hydrogels, which are widely investigated for application in tissue engineering using 3D printing and 3D bioprinting fabrication techniques. The major challenge of using hydrogels for tissue fabrication is their lack of regeneration ability, uncontrolled swelling, degradation and inability to hold 3D structure on their own. Free hydroxyl groups on the surface of SiO_2_ nanoparticles have the ability to chemically interact with alginate–gelatin polymer network, which can be explored to achieve the above parameters. Hence validating the incorporation of SiO_2_ nanoparticles in a 3D printable hydrogel polymer network, according to the patient's critical defects has immense scope in bone tissue engineering. In this study, SiO_2_ nanoparticles are loaded into alginate–gelatin composite hydrogels and chemically crosslinked with CaCl_2_ solution. The effect of SiO_2_ nanoparticles on the viscosity, swelling, degradation, compressive modulus (MPa), biocompatibility and osteogenic ability were evaluated on lyophilized scaffolds and found to be desirable for bone tissue engineering. A complex irregular patient-specific virtual defect was created and the 3D printing process to fabricate such structures was evaluated. The 3D printing of SiO_2_ nanoparticle hydrogel composite ink to fabricate a bone graft using a patient-specific virtual defect was successfully validated. Hence this type of hydrogel composite ink has huge potential and scope for its application in tissue engineering and nanomedicine.

## Abstract 2

3D printing of a complex and irregular virtual defect using SiO_2_ nanoparticle and hydrogel composite ink for patient specific defect fabrication.

## Graphical abstract

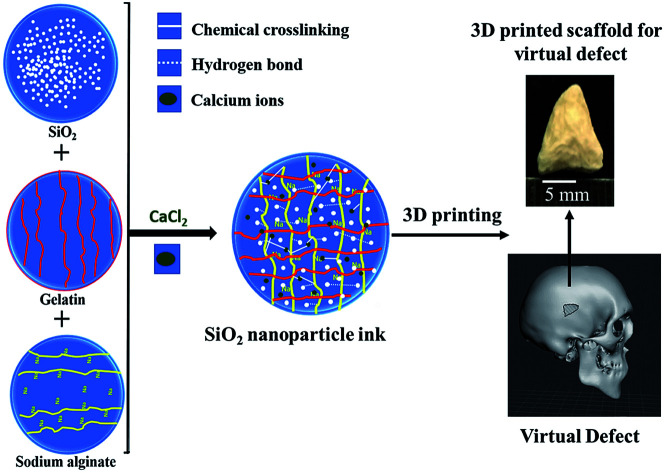

## 1. Introduction

3D printing is receiving huge attention from the whole world due to its high efficiency & precision for product development.^1^ In recent years, this technology has been widely applied in the fields of automobiles, aerospace, the food industry and medical sciences.^2^ Due to its high precision, 3D printing has a huge demand in medical science to develop reusable bio-instruments, patients-specific prosthetic and soft/hard tissue implants.^2^ Patient-specific implants are essential to obtain a facile customized fit in to the defect site with greater accuracy. This technology involves a large amount of preoperative planning from the surgeon depending on the CT or MRI scans of the patient.^3^ Hence surgeons can plan for the better alignment of the implant in the defect site with greater accuracy. Layer by layer deposition of the materials into complex anatomical shapes from a 3D CAD model generated using CT/MRI scans is the main objective of 3D printing for patient-specific medicinal applications of fabricated tissues.^4^ Polymers, ceramics, and metals have been successfully 3D printed for various biomedical applications using different 3D printing technologies like stereolithography (SLA), digital light processing (DLP), fused filament fabrication (FFF) and direct ink writing (DIW) *etc.* Extrusion 3D printing is a variant of fused filament fabrication technique where ceramics or polymers are extruded from a nozzle of a specific diameter into required 3D designs.^5^ Polymer hydrogels like alginate, gelatin, chitosan, *etc.*, are vastly investigated for 3D printing and 3D bioprinting purposes using various crosslinking mechanisms like ionic, temperature, pH, photo crosslinking, *etc.*, for their application into tissue engineering.^6–8^

Sodium alginate is a biocompatible & biodegradable natural polysaccharide, which is widely used as cell-laden hydrogel for bio-printing of engineered bone tissues.^9^ Due to less cell reorganization peptides (RGD peptides), sodium alginate has a lack of cell adhesion sites and limited cell functioning.^7^ Gelatin is another natural biomaterial which is highly used for tissue engineering applications. It a collagen derived polymer with a high number of RGD sequences that facilitate cell adhesion.^10^ Alginate is usually crosslinked with CaCl_2_ whereas, gelatin provides low temperature (4–14 °C) gelation effect and undergoes temperature dependent crosslinking mechanism. Hence at normal physiological temperature gelatin faces critical challenges with respect to crosslinking. In order to achieve a simple and facile mode of cross-linking mechanism, gelatin is often used in combination with various other polymers. Usually, methacrylate polymers are used for photo crosslinking and alginate is used for covalent and ionic crosslinking.^11^ The combination of composite hydrogels using alginate and gelatin polymers show good biocompatibility as oxidized alginate and gelatin undergo covalent bonding and can be ionically crosslinked. Gelatin provided the required RGD (Arg-Gly-Asp) peptides which facilitate enhanced cell adhesion property. Biofabrication of tissue grafts using 3D printing with alginate and gelatin polymers still faces challenges as a very high concentration of alginate and gelatin are required to achieve the required viscosity, mechanical strength, and porosity. Achieving a certain level of micro porosity less than 100 μm, which is a crucial parameter for cell adhesion and proliferation is still a challenge. Hence an alternate mechanism is required to achieve the required viscosity, mechanical strength, and porosity. Bioceramics like SiO_2_ nanoparticles are used in combination with various polymers as a composite material to improve the mechanical strength of the polymers.^12^ SiO_2_ nanoparticles have free –OH groups on their surface which have strong affinity to form a hydrogen bond with COO– groups present in biopolymers like sodium alginate, gelatin, agar, *etc.*^13^ In addition, it can be used for addition of growth factors or other bioactive molecules. Formation of a new hydrogen bond improves mechanical strength and increases the viscosity of hydrogel.^14^ A recent study reported that SiO_2_ nanoparticles promotes osteo-conduction, improves osteoblast proliferation and induce osteogenic differentiation.^15,16^ The release of Si^4+^ ions from SiO_2_ nanoparticles are also reported to enhance angiogenic ability of human endothelial cells.^17^ Hence incorporating silica nanoparticles into alginate and gelatin hydrogels appear to be a promising solution to achieve the required viscosity and mechanical strength for the 3D printed structures. Finally, by lyophilizing the 3D printed structures the required level of micro porosity can be obtained and even the shape of the scaffolds can also be maintained for easy handling of grafts during implantation. Moreover, SiO_2_ on its own has a high potential in health care and medical industry due to its ability to carry various regenerative and cancer drugs. Validation of a 3D printing process of silica nanoparticles for bone tissue engineering application is not yet reported.

In this study, SiO_2_ nanoparticles are loaded into alginate–gelatin composite hydrogels and chemically crosslinked with CaCl_2_ solution. The effect of SiO_2_ nanoparticles on the viscosity, swelling, degradation, compressive modulus (MPa), biocompatibility and osteogenic ability are evaluated on lyophilized scaffolds. A complex irregular patient-specific virtual defect is created and the 3D printing process to fabricate such structures is evaluated.

## 2. Materials & methods

### 2.1 Materials and methods

Sodium alginate and gelatin purchased from HIMEDIA, Hyderabad, India. Calcium chloride was obtained from SD Fine Chem. Limited, India. 10× PBS (Sigma Aldrich, India) was diluted to 1× PBS and used in experiments. For the synthesis of SiO_2_ nanoparticles, reagents like tetraethyl orthosilicate (TEOS, 99%, Alfa Aesar), ethanol (99.98%, Pharmco-Aaper) and ammonia (30%, Sisco Research Laboratories) were used.

#### 2.1.1 Synthesis of SiO_2_ nanoparticles

SiO_2_ nanoparticles were synthesized using Stöber process, under basic conditions. In brief, the synthesis was carried out by mixing 1.33 g of TEOS in 5.5 g ethanol and allowed to stir at room temperature for 5 min. Later, a solution containing 5.5 g of ethanol, 0.5 g DI water, and 0.544 g NH_4_OH was added. The reaction was allowed to continue for 1 h at room temperature. Next, the reaction mixture was filtered and washed thoroughly with water and ethanol to obtain a solution with neutral pH and was dried overnight at 60 °C.^18,19^

#### 2.1.2 Preparation of hydrogel

Alginate/gelatin/SiO_2_ (AGS) hydrogels were prepared by varying the concentration of SiO_2_ nanoparticle (0%, 2.5% and 7.5%) (w/v) and were named as group A, group B and group C respectively and used throughout the manuscript for better understanding. For this purpose, 2.5% (w/v) of sodium alginate was mixed with different concentrations of SiO_2_ nanoparticles dispersed in water followed by stirring at room temperature until a homogeneous solution was obtained. 8% (w/v) of gelatin was added to the above solution under continuous stirring at 60 °C for 1 h.^20^ The compositions of SiO_2_ nanoparticles, sodium alginate and gelatin were listed in Table 1.

**Table 1:** Table showing sample compositions, number of SiO_2_ nanoparticles, viscosity (Pa s), swelling (wt%), degradation (wt%), compressive modulus (MPa) and 3D printability of sample groups with different SiO_2_ concentrations

Sample group	Sodium alginate (w/v%)	Gelatin (w/v%)	SiO_2_ (wt%)	Number of SiO_2_ nanoparticles	Viscosity (Pa s) (at shear rate 10 s^−1^)	Swelling wt% (after 72 h in PBS)	Degradation wt% (after 72 h in PBS)	Compressive modulus (MPa)	3D printability
Group A	2.5%	8%	0%	0	2.28	1268.24 ± 30.08	61.05 ± 4.26	32.57 ± 0.98	No
Group B	2.5%	8%	2.5%	8.92 × 10^15^	16	1204.59 ± 16.38	57.18 ± 1.35	39.49 ± 2.76	Yes
Group C	2.5%	8%	5%	17.85 × 10^15^	13.65	998.27 ± 87.54	54.81 ± 0.89	49.18 ± 1.64	Yes

#### 2.1.3 Preparation of lyophilized scaffolds

*In vitro* tests like swelling, degradation and compression were performed on lyophilized hydrogel scaffolds prepared by a freeze casting method. Polyethylene cylindrical tube with diameter 5 mm, was filled with the prepared hydrogel and was frozen at −20 °C for 24 h. The frozen hydrogel was slowly extruded using a plunger and was cut into small uniform discs of height 5 mm with a surgical blade and chemically cross-linked using CaCl_2_ (10 M) solution for 15 min. Crosslinked scaffolds were again frozen at −20 °C for overnight and lyophilized for 24 h to form porous scaffolds.

### 2.2 Physico-chemical characterization

#### 2.2.1 FT-IR spectroscopy

The lyophilized scaffolds were crushed into fine powders and Fourier transform infrared (FT-IR) analysis of 0.1 g of powder samples was performed with a Tensor 37 FTIR spectrometer system (Bruker Optics, Ettlingen, Germany) equipped with OPUS software (v.6.0 Bruker Optics, Ettlingen, Germany) for spectral acquisition and instrumental control. Infrared spectra were obtained in the range between 4000 and 400 cm^−1^ at a data acquisition rate of 4 cm^−1^ and by maintaining the working temperature at 25 °C.

#### 2.2.2 Inductively coupled plasma-mass spectroscopy (ICP-MS)

The mass percentage of silicon (Si 28) isotope in the scaffolds group B and group C with 2.5 and 5 wt% SiO_2_ nanoparticles concentration was measured by induction coupled plasma-mass spectroscopy (ICP-MS, Bruker). The scaffolds were digested in 5 ml of HNO_3_ and the volume was made to 30 ml with deionized water and 0.5 ml of the digested scaffold solution was further diluted to 25 ml using deionized water and used for ICP-MS analysis.

#### 2.2.3 Surface morphology and rheology

Surface morphology of SiO_2_ nanoparticles was studied using Hitachi S-3400 scanning electron microscope (SEM) operating at 20 kV accelerating voltage and 4.9 mm working distance. The synthesized SiO_2_ nanoparticles were first dispersed in ethanol and drop cast on the sample stub. After drying, the sample was gold sputtered to get a thin conductive layer. Surface morphology of lyophilized scaffolds without cells was examined by SEM (Supra 40, ZEISS) at an accelerating voltage of 5 kV and a working distance of 12 mm. All scaffolds were sputter coated with 5 nm gold film before SEM was performed. Rheology of different AGS hydrogels was analyzed by rheometer (Anton paar, MCR 72) with a shear rate of 0.01 at room temperature.

#### 2.2.4 Swelling and degradation in PBS

*In vitro* tests like swelling and degradation of lyophilized scaffolds were performed in PBS (1×, Sigma Aldrich). In a 6 well plate, lyophilized scaffold with diameter 10 mm and thickness 5 mm was stored in 10 ml 1× PBS at 37 °C for 72 h. The swollen scaffolds were gently washed with deionized water and gently blotted with a tissue paper to remove the external adsorbed liquid and weighed. Swelling weight percent is calculated as
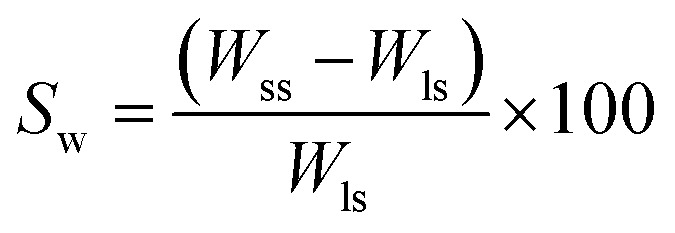
where, *W*_ss_ = weight of swollen scaffold after 72 h, *W*_ls_ = weight of lyophilized scaffold.

To study the degradation behavior, swollen scaffolds were lyophilized for 24 h and the lyophilized scaffolds were weighed. Degradation weight percent is calculated as
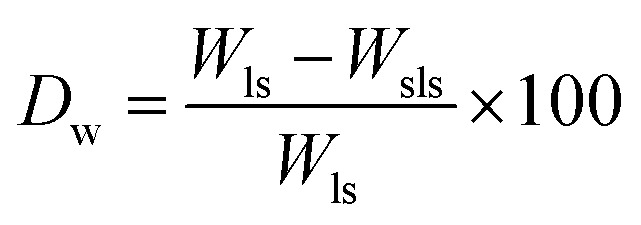
where, *W*_ls_ = weight of lyophilized scaffold, *W*_sls_ = weight of swelled lyophilized scaffold.

#### 2.2.5 Mechanical testing of lyophilized scaffolds

Compression test was performed on lyophilized scaffolds (diameter 5 mm & height 10 mm) with the help of UTM (Universal testing machine, Instron 5900 series). For all lyophilized scaffolds, length (*L*) and diameter (*D*) was measured with a Vernier caliper before the compression test. The load of 10 kN and strain rate 1 mm min^−1^ was set during the test. Stress and strain were calculated as
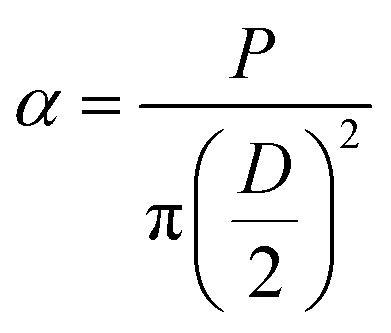

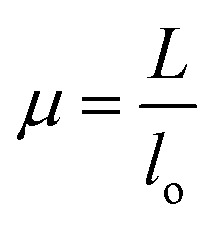
where *α*, *P*, *D*, *L*, *μ*, and *l*_o_ denote stress (MPa), *L*_load_ (N), *D*_diameter_ (mm), *L*_length_ (mm), strain, and gauge length respectively. Compressive modulus was then calculated form stress–strain curves using methods previously reported.^21^

### 2.3 Cell studies

*In vitro* biological tests of lyophilized scaffolds were carried out with human umbilical cord mesenchymal stem cells (UMSCs). UMSCs were isolated from umbilical cord of a healthy adult female donor during childbirth.^22^ The experimental procedure was accepted by the Institutional Ethics Committee (IEC), IIT Hyderabad (Indian Institute of Technology Hyderabad) in accordance with the guidelines of ICMR-DBT for stem cell research 2017, India and informed consent was obtained from the patient. The isolated and cultured umbilical cord-derived MSCs successfully differentiated into trilineage differentiation as described before.^23,24^ They were cultured in T75 flasks (Corning, India) using DMEM (Dulbecco's modified Eagle's medium, Sigma-Aldrich, India) supplemented with 10% FBS (fetal bovine serum, Sigma-Aldrich, India), 1% l-glutamine and 1% antibiotic-antimycotic solution (penicillin–streptomycin, Invitrogen, Thermo Fischer, India) and maintained at 37 °C with the supply of 5% CO_2_ and 95% humidity in a CO_2_ incubator (Thermo Scientific Forma series-3131, India). The nutrient medium was changed for every 48 h. Adherent cells were trypsinized (0.25% trypsin–EDTA, Sigma-Aldrich, India) after reaching 70–80% confluency and sub cultured until passage 5. For the entire cell culture experiments, cells with passage 5 were used directly after trypsinization. 50 000 cells for 50 μl of media were seeded on each scaffold in a 24 well plate (Corning, India) and maintained at 37 °C with the supply of 5% CO_2_ in a CO_2_ incubator. The medium was changed for every 24 hours during the complete study.^23^

#### 2.3.1 Alamar blue and live/dead cell assay

The scaffolds were sterilized overnight in a laminar air flow chamber using 70% ethanol followed by UV sterilization for an hour. Alamar blue dye reduction assay (Bio Source International, Camarillo, CA, USA) was performed to determine the metabolic activity of the UMSCs on day 1, 7, 14 and 21 as described previously.^25,26^ Absorbance at 570 nm and 600 nm was recorded by a microplate reader (Enspire® multimode plate reader, PerkinElmer, MA, USA) and percentage of dye reduction was calculated. FDA (fluorescein diacetate, Invitrogen, India) 2 μg ml^−1^ and 20 μg ml^−1^ PI (propidium iodide Invitrogen, India) in 1× PBS are used as fluorescent dyes for tagging live cells with green and dead cells with red respectively. Fluorescence microscopy images were obtained for all the three groups of scaffolds using a fluorescent microscope (Apotome 2, Carl-Zeiss, Germany) on day 1 and day 7. The assay was performed according to the manufacturer's protocol and scaffolds with FDA dye solution were incubated for 20 min at 37 °C in a CO_2_ incubator and 5 min at room temperature for PI dye solution. After incubation scaffolds were gently washed with 1× PBS and viewed under microscope.^24^

#### 2.3.2 Cell proliferation and differentiation

The cell proliferation was evaluated by measuring the quantity of dsDNA using pico green assay on day 1, 7, 14 and 21 respectively as described previously.^26^ The scaffolds were lysed using lysis buffer (10 mM tris, pH 7.0, 1 mM EDTA and 0.2% v/v Triton X-100; all from Sigma-Aldrich, USA). Afterwards, 100 μl of pico green (Molecular Probes, Invitrogen GmbH, Karlsruhe, Germany) at 200× dilution in TE buffer was added to 50 μl of the sample and incubated at room temperature for 5 min without exposing to light. Excitation and an emission wavelength of 485 nm and 520 nm was used to measure the fluorescence intensity using a microplate reader (Enspire® multimode plate reader, PerkinElmer, MA, USA).

### 2.4 3D printing of patient-specific scull defect

Patient-specific CT scan of a healthy adult was obtained from the hospital (MNR hospital, Hyderabad, India) with the consent of the patient. As only a virtual osteotomy was performed no ethical approval was required. The obtained scans were converted to DICOM images using InVesalius 3.1 (© 2007–2017 Center for Information Technology Renato Archer CTI) software. An image of 11 mm length and 11 mm breadth was virtually created in an irregular fashion as shown in Fig. 6 and exported into a STL file using “slicer” and “meshmixer” software. Slicing of STL file and G-code conversion were done using “Repetier host” software and 3D printed using BIOBOT (Allevi) 3D printer. In brief, the 3D printing was performed by loading the prepared hydrogel inks into a syringe and extruded with a pressure of 35 Psi at a printing speed of 10 mm s^−1^. The infill density was kept 100% during the printing of the virtual defect model.

### 2.5 Statistical analysis

Results are presented as mean ± standard deviation. GraphPad Prism software (GraphPad Software, San Diego, CA, USA) was used to perform statistical analysis for all the results with *n* = 3. A one-way analysis of variance (ANOVA) followed by Bonferroni's *post hoc* test was used to extract the level of statistical significance. *P* values < 0.05 were considered statistically significant at a confidence level of 95%.

## 3. Results

**Fig. 1:**
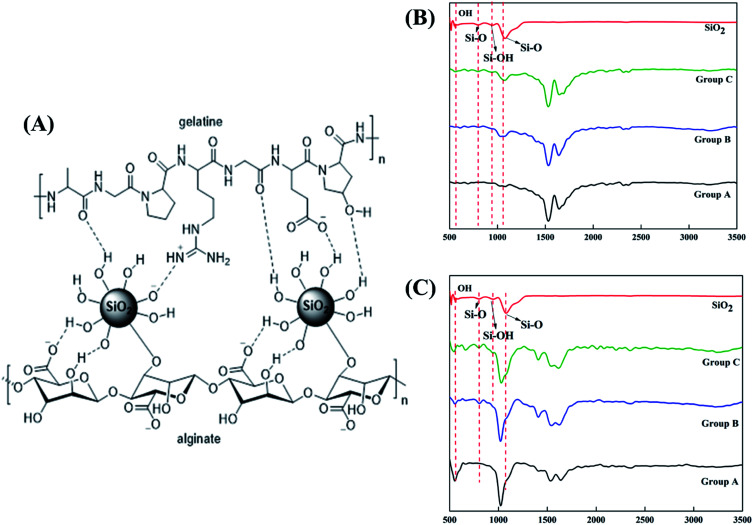
Possible mechanism in of SiO_2_ integration in the alginate–gelatin hydrogel system (A), FTIR spectra of lyophilized hydrogels (B) and FTIR spectra of lyophilized hydrogels after 72 hours immersion in PBS (C) respectively.

**Fig. 2:**
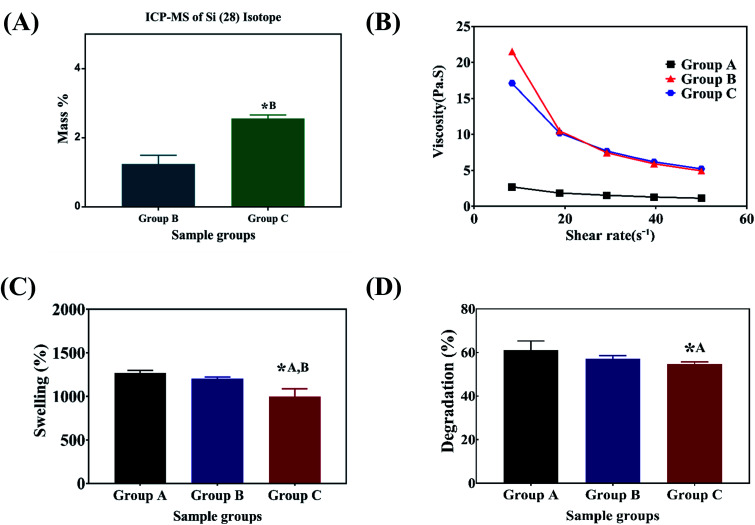
Viscosity of the hydrogels used for 3D printing (A), swelling percentage (B), degradation percentage (C) and mass percentage calculated using ICP-MS (D) of the lyophilized hydrogels. * indicates a significant difference between sample groups with *P* < 0.005.

SiO_2_ nanoparticles were successfully integrated into the alginate–gelatin hydrogel system. Fig. 1A depicts the possible mechanism of SiO_2_ nanoparticles integration in the alginate–gelatin hydrogel system forming a SiO_2_ nanoparticle ink. To confirm the chemical structure of the functional groups and to check the purity of the prepared samples, FTIR spectra of the lyophilized hydrogels and the lyophilized hydrogels after 72 hours immersion in 1× PBS are shown in Fig. 1A and B respectively. The absorption band at around 799 cm^−1^ is arising from the symmetric vibration of the Si–O bond. The band appearing at 942 cm^−1^ is assigned to the asymmetric vibration of Si–OH. The band at around 1080 cm^−1^ corresponds to the asymmetric stretching vibration of the Si–O–Si bond.^17,27^ All the bands apart from the characteristic bands of SiO_2_ can be attributed to the characteristic bands of alginate and gelatin. The bands at 1645, 1535 and 1243 cm^−1^ were identified to the C=O vibration, bending modes of C=N and N–H vibration respectively. The characteristic bands of sodium alginate appearing at 1312 and 1413 cm^−1^ were assigned to the asymmetric and symmetric stretching of –COO groups, respectively. The strong bands at 1413 cm^−1^ in samples after immersion into PBS (Fig. 1C) correspond to the symmetric vibrations of C=O.^13,14,28^ The bending modes observed at 1020 cm^−1^ correspond to the (PO_4_)^3−^ bending mode indicating the precipitation of phosphate from phosphate buffer.^29^ The elemental concentration of silicon (Si 28) isotope in the scaffolds with 2.5 and 5 wt% addition of SiO_2_ nanoparticles was found to be 12.41 ± 2.55 and 25.59 ± 1.01 g kg^−1^ respectively. Fig. 2A shows the mass percentage of silicon in scaffold groups B and C. ESI 1† shows the SEM image of SiO_2_ nanoparticles at different magnifications. It was observed that particles are spherical in morphology. Average particle size was calculated using Image J software and it was found to be 64 ± 8.9 nm. The number of SiO_2_ nanoparticles in all the sample groups calculated using SEM images are presented in Table 1. The calculations used for the same are described in ESI 1.† The maximum SiO_2_ content could not exceed 7.5 wt% by *in situ* synthesis; beyond this concentration, phase separation occurred, and a uniform and homogenous gel could not be obtained. The viscosity of all the sample groups is shown in Fig. 2B. As SiO_2_ nanoparticle concentration increases in the alginate–gelatin hydrogel system, their viscosity gradually increases up to 5% of SiO_2_ nanoparticle concentration at a shear rate of 10 s^−1^. For group B with 2.5% SiO_2_ nanoparticle concentration, the viscosity is the highest. There after even with the increase in the SiO_2_ nanoparticle concentration viscosity remains approximately same up to 5% SiO_2_ concentration and found to be decreasing with a gradual addition up to 7.5% (ESI 2†).

**Fig. 3:**
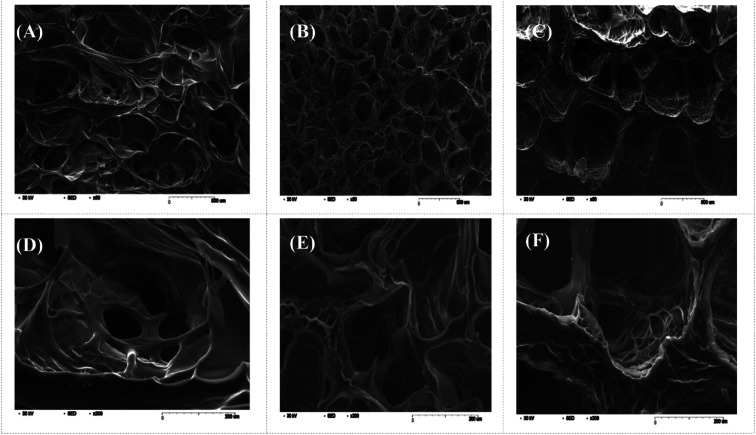
SEM images of the lyophilized hydrogels (A), (B) and (C) indicate sample groups A, B and C respectively at scale bar 500 μm and (D), (E) and (F) indicate higher resolution images of sample groups A, B and C respectively at scale bar 200 μm.

Swelling & degradation percentage of lyophilized scaffold after 72 hours immersion in 1× PBS at 37 °C are represented in Fig. 2C and D respectively. With increasing SiO_2_ nanoparticle concentration, swelling reduces. Swelling is minimum for group C. Degradation was also found to be reduced with increasing in SiO_2_ nanoparticles concentration (Table 1). Viscosity, swelling and degradation property of the prepared group C hydrogel is compared with the commercially available bioinks from vendors like Cellink (Cellink, Sweden), BioInk (RegenHU, Switzerland) and Bio-Gel (BioBots, US) and is presented in Table 2. Group C shows better viscosity, swelling and degradation when compared with nanocellulose based bioink provided by Cellink. Scanning electron micrographs of the lyophilized scaffolds of all sample groups are shown in Fig. 3. The surface morphology of the scaffolds at lower magnification indicates that group B has a smaller pore size compared to that of group A and group C, however, micro porosity is profound in group C when compared to group A and B. When the SiO_2_ addition in the hydrogel system reaches beyond 5 wt% *i.e.*, at 7.5 wt%, the nanoparticles tend to agglomerate and are precipitated on the surface of the lyophilized samples as evident from ESI 2.† The gradual increase in SiO_2_ nanoparticle concentration from group A to group C increases the compressive modulus of the lyophilized scaffolds. The compressive modulus (MPa) of the samples of all groups are statistically significant (*P* < 0.001). Sample groups A, B, and C are subjected for biocompatibility tests using FDA/PI live dead staining (Fig. 5), Alamar blue dye reduction assay (Fig. 6A) and pico green total DNA quantification assay (Fig. 6B). FDA/PI stained fluorescence micrographs of day 1 indicate the cell attachment on the surface of the scaffolds of all sample groups but cells are more rounded in group A and B resembling cells embedded in a typical hydrogel. Attached cells on the surface of group C show more protrusions when compared to group A and B. FDA/PI images obtained on day 7 indicate the proliferation of cells in all sample groups and are more predominant in group C.

**Table 2:** Table showing the viscosity (Pa s), swelling% and degradation% of commercially available bioinks and the prepared hydrogel (group C with 5 wt% SiO_2_ nanoparticle concentration)

Company	Bioink	Materials	Viscosity (Pa s)	Swelling%	Degradation%	Ref.
CELLINK	CELLINK	1.36% nanocellulose and 0.5% alginate crosslinked with cationic solution	11 ± 0.7	1145 ± 42	70 ± 5	39–41
RegenHU	BioInk®	Polyethyleneglycol-diacrylate (PEGDA) photo-crosslinked with photoinitiator	1.05 ± 0.09 (100 wt% PEGDA)	342 ± 3 (100 wt% PEGDA)	53.56 ± 6.16 (100 wt% PEGDA)	39, 40, 42 and 43
Biobot	BioGel	10% gelatin methacrylate photo-crosslinked with 0.05% Irgacure I2959	65 ± 14	719 ± 24	30 ± 2	39, 40 and 44
Group C	As prepared	Alginate/gelatin/SiO_2_ nanoparticle based	13.65 ± 3	998.27 ± 87.54	61.05 ± 4.26	

**Fig. 4:**
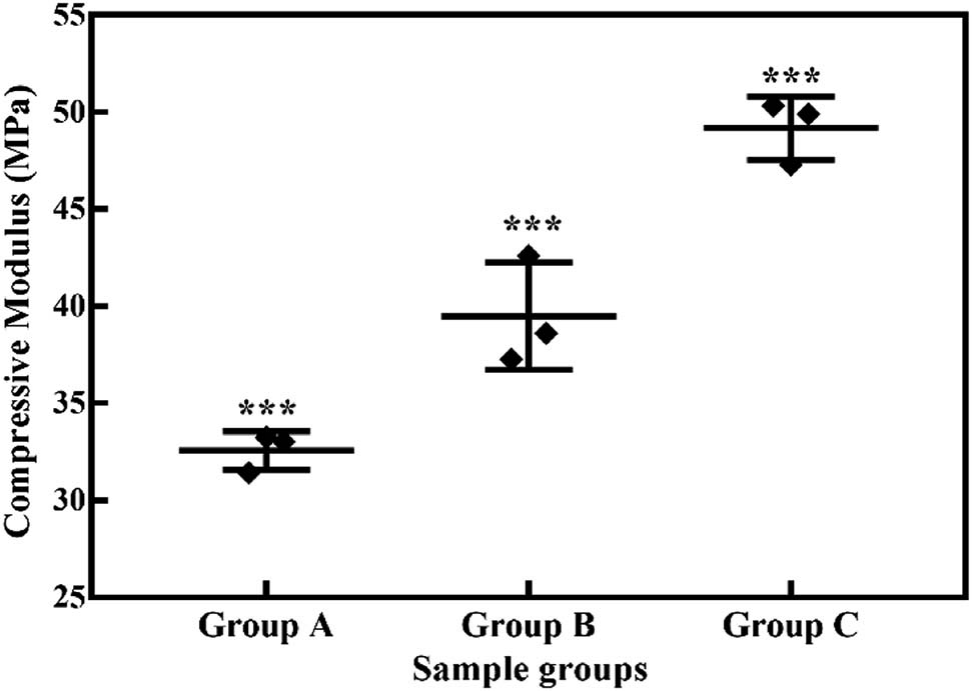
Compressive moduli (MPa) of lyophilized hydrogels. *** indicates statistical significance with *P* < 0.001.

**Fig. 5:**
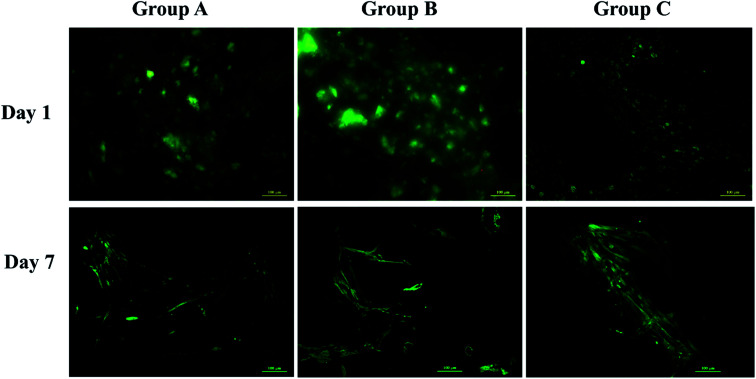
Live dead fluorescent microscopy images of UMSC's cultured on different samples on day 1 and day 7 using FDA (fluorescein diacetate) stained with green and PI (propidium iodide) stained with red.

**Fig. 6:**
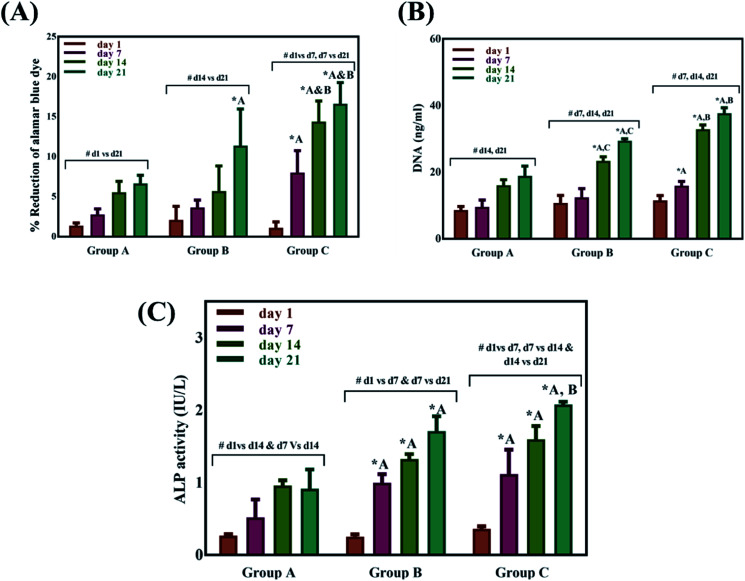
Alamar blue dye reduction% of different sample groups (A), total DNA quantification using pico green assay of different sample groups (B), ALP activity (IU L^−1^) of different sample groups cultured with UMSCs for 21 days (C). * indicated a significant difference between sample groups at same time point with *P* < 0.05 and ^#^ indicates a significant difference between different time points within the same group with *P* < 0.05.

**Fig. 7:**
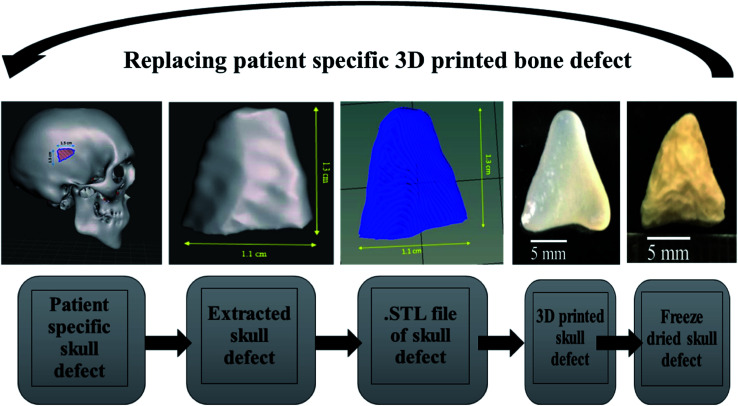
Process showing the 3D printing of patient-specific virtual bone defect.

The Alamar blue dye reduction assay performed for days 1, 7, 14 and 21 indicate the significant increase in metabolic activity of the cells in all sample groups between all the time points from day 1 to day 21. There is no statistical significance in the metabolic activity of the cells seeded on scaffolds between all sample groups after day 1 indicating the same cell seeding density on the surface of the scaffolds. There is no significant difference in the metabolic activity of group A and group B until day 14 but the metabolic activity of group B is significantly different (*P* < 0.05) from group A on day 21. The metabolic activity of group C is significantly higher when compared to group A on all time points from day 7 to 21. There is no statistical significance between group B and group C on day 7 but group C shows a significant increase in metabolic activity compared group B on day 14 and day 21. Total DNA quantification performed on cell seeded scaffolds of all sample groups from day 1 to day 21 are in close agreement with the results obtained from Alamar blue dye reduction assay. There is a significant increase in the DNA content of all sample groups between different time points from day 1 to day 21. The total DNA content of group C sample from day 7 to day 21 is significantly higher when compared to group A. The DNA content of group C as observed on day 14 and day 21 is statistically significant when compared with the DNA content of group B. The alkaline phosphatase activity (Fig. 6C) was analyzed for all the sample groups to study the differentiation of UMSCs into osteogenic lineage. ALP activity (IU L^−1^) of group A samples show a significant difference from day 1 to day 14 but day 14 and day 21 are not significant. ALP activity of group B samples shows a significant increase from day 1 to day 7 and day 7 to day 21. Whereas for group C samples the ALP activity is increasing for all time points from day 1 to day 21 when compared among them. The statistical significance between the groups shows that group B shows increased ALP activity than group A from day 7 to day 21. Group C exhibited a significant increase in ALP activity (IU L^−1^) when compared with the ALP activity of both group A and group B from day 7 to day 21. A virtual skull defect as depicted in Fig. 7 was successfully 3D printed using the formulated nanoparticle ink and the 3D printed defect was subjected for lyophilization. The lyophilized structure was similar to that of the 3D printed structure and to the designed CAD model of the virtual defect. No major change in the external structure with respect to volume was observed after lyophilization.

## 4. Discussions

The major challenge in using hydrogels for tissue fabrication is their lack of regeneration ability, usage of chemicals like CaCl_2_ for crosslinking and their ability to hold 3D structure on their own. The regeneration ability of hydrogels can be increased by loading such hydrogels with various regenerative drugs and growth factors. But controlling the release of such drugs and growth factors is still a challenge and can be rectified by using carries such as silica nanoparticles.^16,30^ Hence validating the incorporation of SiO_2_ nanoparticles in hydrogel polymer network which can be 3D printable according to patient's critical defects has immense scope in bone tissue engineering. In this study, we have shown successfully incorporated SiO_2_ nanoparticles in a 3D printable hydrogel polymer network and validated the process for patient specific defect 3D printing. The viscosity of hydrogels is an important parameter for extrusion-based 3D printing and is expected to increase with the addition of ceramic particles like SiO_2_ nanoparticles.^31^ Hence with the gradual addition of up to 2.5 wt%, the viscosity of the hydrogels increased gradually. But after 2.5 wt% up to 5 wt% addition, the viscosity remained almost same indicating the saturation and phase separation hence reduction in resistance.^32^ Thereafter, when the concentration reached to 7.5 wt% the viscosity appeared to be lower than 2.5% (data not shown). After a certain addition of silica, they tend to agglomerate and lose their colloidal property hence start to settle at the bottom of the hydrogel suspension. Once the homogenous dispersion of SiO_2_ nanoparticles in the hydrogel suspension is lost they are deemed to be not suitable for 3D printing hence 2.5 wt% and 5 wt% silica incorporated hydrogels were selected for further analysis. ICP-MS analysis also confirms the increase in silicon elemental concentration with addition of SiO_2_ nanoparticles in the hydrogel system. As the SiO_2_ nanoparticle concentration in the alginate–gelatin hydrogel mixture increases, the free OH groups present on the surface of SiO_2_ facilitate more bonding sites for the formation of hydrogen bond between SiO_2_ and sodium alginate as well as SiO_2_ and gelatin.

As evident from the FTIR spectrum all the peaks corresponding to SiO_2_ nanoparticles are present in group B and group C which are clearly absent in group A (Fig. 1B and C). This indicates the presence of SiO_2_ nanoparticles in the hydrogel network. Even after 72 hours immersion in PBS, the peaks corresponding to SiO_2_ are quite evident. The Si–O group at 1080 cm ^−1^ and C–O–C group at 1070 cm^−1^ are merging together indicating hydrogen bonding between silica and alginate–gelatin polymer network. Therefore, the active sites facilitating the binding of water molecules are also reduced due to the hydrogen bonding with SiO_2_. This may be correlated to the decrease in swelling percentage of the hydrogel system with an increase in the SiO_2_ concentration. Swelling is also used to determine the extent of crosslinking. More degree of swelling results in less crosslinking and *vice versa*. In this case, though all the hydrogel groups are crosslinked using CaCl_2_ solution for the same duration, the hydrogels with more silica concentration exhibit less swelling. This indicates the additional degree of crosslinking achieved by the hydrogen bonding between silica and alginate–gelatin polymer network. The extent of crosslinking also determines the rate of degradation, hence the results of swelling and degradation correlate with each other verifying the interaction of SiO_2_ and alginate–gelatin polymer network. Swelling and degradation properties of hydrogels also have an important role to play in tissue engineering the water retaining ability and the degradation are to be controlled to achieve a controlled release of drugs, growth factors and ions.^28,33^ Using SiO_2_ nanoparticles as an additional crosslinking agent appears to achieve this objective of controlling the swelling and degradation properties of the alginate–gelatin hydrogels. With the inclusion of ceramic nanoparticles into the polymer hydrogel, these hybrid composite materials (group B and C) are expected to show enhanced mechanical properties when compared with the normal polymer ink (group A) as evident from Fig. 4. The increased compressive modulus (MPa) for group B and group C samples may also be due to the tight bonding of silica with the free OH^−^ and COO^−^ functional groups in the alginate and gelatin polymer network. The lyophilized scaffolds exhibit similar macro porosity across all groups but, appears to be slightly more in group B. Whereas, micro porosity is appeared to be significantly more in group C as observed from higher magnification SEM images. This may be due to the pattern of water accumulation during the process of gelation. As group B has higher viscosity and even distribution of SiO_2_ nanoparticles, it shows homogeneous gelation resulting in uniform macro porosity. In the case of group C, the hydrogel suspension reaches its maximum capacity to accommodate SiO_2_ nanoparticles and water accumulation is minimum enabling the development of micro porosity on the surface during the process of lyophilization.

The surface morphology of the scaffolds significantly affects the proliferation and differentiation of MSCs. Hence, group C with micro porosity facilitates the adherence of MSCs better than group A and group B by facilitating greater surface area and nutrient infiltration.^34^ The cell viability on the scaffolds is in correlation with the earlier reports suggesting the proliferation of cells as the effect of silica nanoparticles. The significant increase in the DNA content of cells seeded on the surface of group C indicates the proliferation of cells with time from day 1 to day 21. The metabolic activity analyzed using Alamar blue dye reduction assay and total DNA quantification using pico green agree with each other. SiO_2_ nanoparticles are known to promote osteogenesis, the release of silicon ions have a direct impact on promoting osteogenic pathways thereby enhancing osteogenesis.^34,35^ As reported by Shie *et al.*,^34^ Si ion concentration at an appropriate level helps in the proliferation of osteoblast like cells and actively stimulate the production of osteo specific proteins. Hence the ALP activity of group C scaffolds is significantly higher when compared to group A and group B scaffolds. This may be due to the effect of Si ions, as they actively stimulate the entry of cells into S and G2 phases of cell division. SiO_2_ nanoparticles have various applications in tissue engineering and regenerative medicine. The functionalization ability of SiO_2_ nanoparticles makes them an effective carrier for various drugs, growth factors.^30,36–38^ 3D printing as a bio fabrication technique to develop patient-specific bone grafts has taken place in recent years for effective bone regeneration therapies. The SiO_2_ nanoparticles seem to enhance osteogenic ability when incorporated in the alginate–gelatin hydrogel mixture but the validation of the prepared hydrogel ink for 3D printing a patient-specific defect is crucial for tissue engineering application. The virtual irregular large scale defect created on a skull model using a patient CT scan was successfully 3D printed using 5 wt% silica loaded hydrogel ink. This is assumed to show better cell viability and enhanced osteogenic ability as observed from cell proliferation studies and ALP assay. By replacing the SiO_2_ nanoparticles with mesoporous SiO_2_ nanoparticles (MSNs) their ability to deliver regenerative drugs and growth factors can be explored further. Various previous studies have explored the ability of MSNs in controlled release of anticancer drugs by functionalizing them with various bioactive compounds.^37^ This ability of SiO_2_ nanoparticles in synergy with their osteogenic ability offers huge scope for the above validated technique for their use in 3D printed models for bone tissue engineering and drug delivery applications.

## 5. Conclusion

Addition of SiO_2_ nanoparticles into the hydrogel system has increased the viscosity of the hydrogel ink up to a certain concentration of 2.5 wt%, which increased printability of the scaffold. Compressive modulus (MPa) has been significantly improved whereas, swelling and degradation properties are significantly inhibited. Micro porosity favoring cell attachment and proliferation can also be enhanced. Biocompatibility and osteogenic ability of the hydrogels are significantly increased with the addition of SiO_2_. 3D printing of SiO_2_ nanoparticle hydrogel composite ink to fabricate a bone graft using a patient-specific virtual defect was successfully validated. Hence this type of hydrogel composite ink has huge potential and scope for its application in tissue engineering and nanomedicine. This study of validating the 3D printing of SiO_2_ nanoparticles opens the possibility of exploring the use of mesoporous SiO_2_ and functionalizing the nanoparticles with desirable growth factors and drugs. This approach seems to be promising for creating an impact in the health care industry.

## Ethical approval

This article does not contain any clinical study with human participants or animals performed by any of the authors.

## Conflicts of interest

The authors declare that there is no conflict of interest.

## Supplementary Material

RA-009-C9RA03641E-s001
