# Supplementary material for: 3D printable SiO2 nanoparticle ink for patient specific bone regeneration
Source: RSC Adv. 2019 Jul 31;9(41):23832–42. doi: 10.1039/c9ra03641e (PMC9069463; doi:10.1039/c9ra03641e)
Supplement: RA-009-C9RA03641E-s001 [file RA-009-C9RA03641E-s001.pdf]

Electronic Supplementary Material (ESI) for RSC Advances.
This journal is © The Royal Society of Chemistry 2019

Supplementary Information

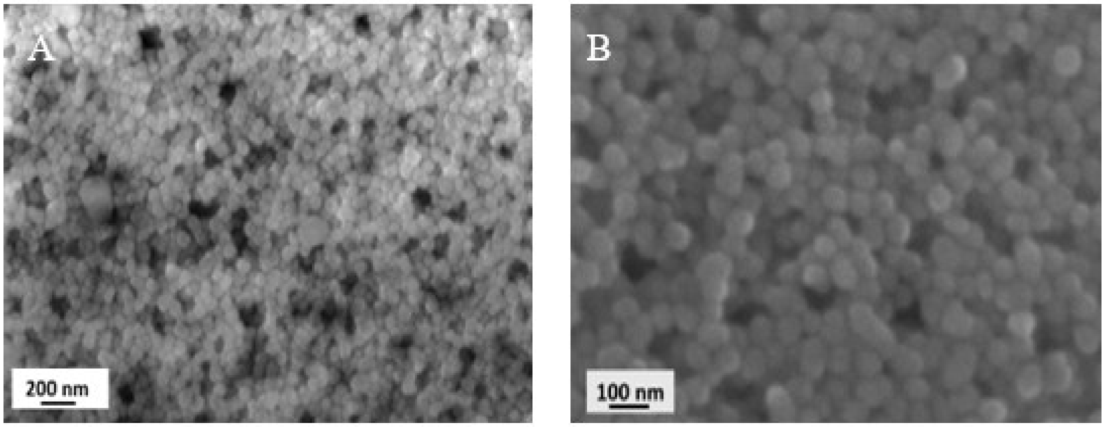

Supplementary Information 1. *SEM image of prepared SiO_2 nanoparticle at scale 200µm (A). SEM image of at SiO_2 nanoparticle at scale bar 100 µm (B)*

With respect to the above SEM image, average particle size of the SiO_2 nanoparticle is 64 nm (calculated from image J software).

Radius of SiO_2 particle, r_{silica}= 32 nm.

Density of SiO_2, ρ_{silica} = 2.05 g/cm^3.

Volume of single SiO_2 particle, $V_{silica} = 4\pi r_{silica}{}^3/3 = 1.37 \text{ X } 10^{-16}$ cm^3.

Weight of a single SiO_2 particle, $W_{silica} = \rho_{silica} \text{ X } V_{silica} = 2.80 \text{ X } 10^{-16}$ g.

No. of SiO_2 particles per gram, $N = 1/ W_{silica} = 3.57 \text{ X } 10^{15}$ particles per gram.

Hence, Group A has 0 SiO_2 particles, Group B has 8.92 X 10^{15} particles and Group C has 17.85 X 10^{15} particles.

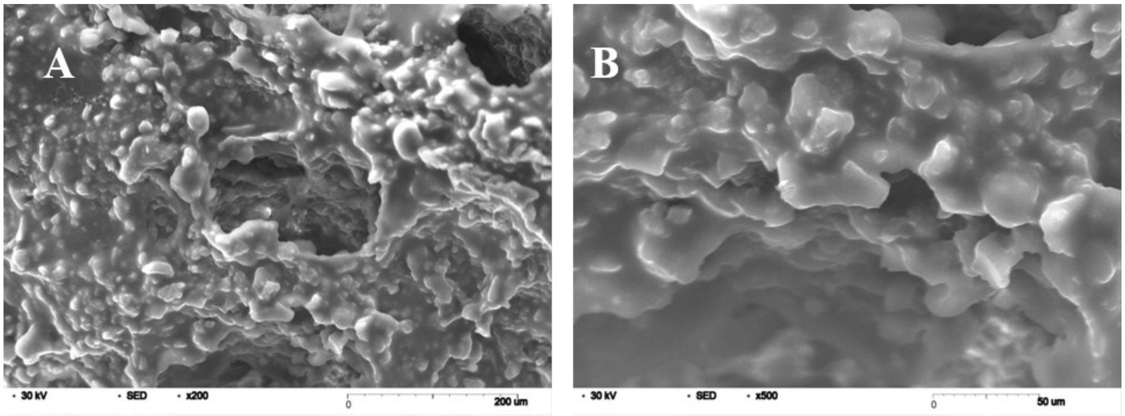

Supplementary Information 2. *SEM image of lyophilized scaffold with 7.5wt % of* SiO_2 *nanoparticle at lower magnification with scale 200µm (A). SEM image of lyophilized scaffold with 7.5wt % of* SiO_2 *nanoparticle at higher magnification with scale bar 50µm (B).*
